# A chromatic-switching Fe_2_Nb_2_O_7_ pyrochlore cage nanozyme for robust peroxidase–mimetic activity and selective hydrogen peroxide detection in complex media

**DOI:** 10.1039/d6ra03158g

**Published:** 2026-07-07

**Authors:** Muhammad Ishfaq Ahmad, Mian Hasnain Nawaz, Akhtar Hayat, Iqra Khalid, Peter John, Muhammad Nasir

**Affiliations:** a Interdisciplinary Research Centre in Biomedical Materials (IRCBM), COMSATS University Islamabad Lahore Campus, 1.5 Km Defence Road, off Raiwind Road Lahore Punjab 54000 Pakistan muhammadnasir@cuilahore.edu.pk; b Government College University Lahore Katchery Road, Anarkali Lahore Punjab 54000 Pakistan peterjohn@gcu.edu.pk; c Department of Chemical Engineering, Faculty of Engineering, Islamic University of Madinah Madinah 42351 Saudi Arabia

## Abstract

Hydrogen peroxide (H_2_O_2_) is a reactive oxygen species (ROS) that plays a role in various physiological processes, with abnormal levels linked to multiple diseases. Nanozymes, which are praised for their easy synthesis, stability, cost-effectiveness, and recyclability, are gaining interest for applications in cancer treatment, disease diagnosis, and molecular sensing. However, their limited catalytic activity and lack of multifunctionality restrict their sensitivity and broader applicability. Therefore, developing highly active, multipurpose nanozymes is essential for enhancing their utilization across various fields. In this work, a novel pyrochlore Fe_2_Nb_2_O_7_ was synthesized through the co-precipitation method with a controlled combination of iron oxide and niobium oxide for H_2_O_2_ detection. Fe_2_Nb_2_O_7_ pyrochlore exhibited a prominent absorption peak at 645 nm in the UV-vis spectrum. Novel pyrochlore Fe_2_Nb_2_O_7_ exhibits strong repeatability, high selectivity, excellent stability, and reasonable reproducibility in H_2_O_2_ detection. Its notable peroxidase-like activity allows for the catalytic breakdown of H_2_O_2_, generating ˙OH radicals that oxidize colorless 3,3°,5,5°-tetramethylbenzidine (TMB) into its blue oxidized form. Its outstanding sensing capabilities are attributed to a large specific surface area, strong electrical conductivity, numerous active sites, distinctive structural features and the synergistic interaction between Fe_2_O_3_ and Nb_2_O_5_. It facilitated a rapid colorimetric assay with a detection limit of 0.62 µM and a linear range of 01–100 µM. The sensor has also been successfully applied to the detection of H_2_O_2_ in normal human serum. In summary, this study offers a cost-effective, sensitive and visually appealing H_2_O_2_ detection technology with a broad range of potential uses.

## Abstract 2

Graphical representation for the detection of hydrogen peroxide without the use of enzymes.

## Graphical abstract

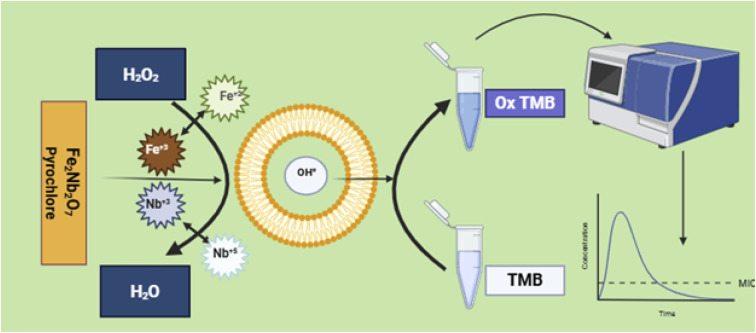

## 1 Introduction

Hydrogen peroxide (H_2_O_2_) plays a crucial role in various biological processes such as vascular remodeling, regulating stomatal closure, and activating immune cells.^1^ It is therefore important in many scientific-technological domains, such as environmental processes, pharmaceutical sciences, mining, nutrition, and medical applications.^2^ However, excessive amounts can have negative consequences such as apoptotic induction, lysosomal membrane rupture, cancer, aging, kidney disorders, cellular damage, and central nervous system diseases.^3,4^ It is crucial to detect hydrogen peroxide effectively. Numerous sensing techniques, such as spectrometry,^5^ chemiluminescence, chromatography, titration^6^ and electrochemistry have been utilized for this purpose. These widely used techniques for H_2_O_2_ detection are costly, time-consuming, and call for qualified analysts. Colorimetric analysis is favored for its low cost, portability, quick response, and ease of use. However, the reliance on natural enzymes poses challenges due to their high cost, sensitivity to pH, limited temperature stability, and vulnerability to degradation. Therefore, a low-cost, highly catalytic, and environmentally resistant natural enzyme replacement must be developed.

Nanozymes are nanomaterials exhibiting enzyme-like activity, attracting attention for their catalytic roles in energy, environmental protection, and catalysis.^7–9^ In addition to their comparable catalytic behavior to natural enzymes, the impact of substrate, temperature, pH, and other external factors on catalysis is also considered,^10^ have several benefits over natural enzymes, including inexpensive cost, adjustable composition, high physiological stability, superior environmental stability, and easily manufactured enzyme mimic functionality.^11–13^ Thus far, over a thousand distinct kinds of nanozymes have been discovered. Molecular detection, illness diagnostics, pollutant degradation, and cancer treatment have all benefited from the application of nanozymes. Various nanomaterials, such as noble metals and metal oxides, exhibit enzyme-like properties,^14–16^ carbon-based nanomaterials,^17^ metal–organic frameworks,^18,19^ single-atom materials.^20,21^

Pyrochlore is a distinct class of materials characterized by a composition akin to A_2_B_2_O_7_. La^3+^, Ce^3+^, Pr^3+^, and Nd^3+^ are examples of trivalent rare earth (RE) ions in this class, especially Fe^3+^ utilized in the pyrochlore structure as a key dopant or constituent to modify magnetic, electrical, and electrochemical properties; Sn^4+^, Ti^4+^, Nb^4+^, Zr^4+^, and Hf^4+^ are examples of tetravalent transition metal ions. Pyrochlore materials have gained attention as sensors due to their exceptional chemical stability, low activation energy, and oxygen defects. By substituting cations/anions of varying valency and ionic size at the A or B and O sites, effective defect sites can be created, enhancing properties for applications in electrocatalysis, photocatalysis, and sensing.

Specifically, ferromagnetic oxide's intrinsic peroxidase activity, the first fascinating finding^22^ Iron oxide nanozymes have garnered significant attention as the most studied nanozymes. They exhibit great potential for application in the domains of cancer treatment, anti-biofouling, magnetic resonance imaging, and biosensing.^23–25^ Because Fe_3_O_4_ has a relatively higher saturation magnetization and easier synthesis methods than Fe_2_O_3_, it has received more attention than the other main iron oxide nanozyme. Nevertheless, Fe_3_O_4_ ferrous ions could increase their hazardous potential and cause chemical instability.^26^ Fe_2_O_3_ nanozymes are likely to be superior choices for such applications. Despite their low enzyme-mimicking activities, these Fe_2_O_3_ nanoparticles are suitable for various applications such as biosensing and antimicrobial therapy. A key challenge remains in producing ultrafine nanoparticles with uniform sizes while preventing aggregation, which can hinder their enzyme-like functionality in catalytic reactions.

Secondly, as an n-type semiconductor, Nb_2_O_5_ finds extensive application in gas sensing, energy storage, solid acid catalysis, and other fields.^27,28^ These investigations have demonstrated a close correlation between Nb_2_O_5_ morphology and surface characteristics and its performance. In particular, Nb_2_O_5_ in the form of nanorods performed better in many applications, such as humidity sensors and dye-sensitized solar cells.

According to a recent report by Tsunehiro Tanaka *et al.*^29^ Nb_2_O_5,_ synthesized *via* a solvothermal reaction and possessing a nanoplate morphology, demonstrated increased catalytic activity for benzyl alcohol specific photooxidation. Hetero-structured hybrids of Nb_2_O_5_ with graphene or reduced graphene oxide have been infrequently explored, in contrast to TiO_2_-graphene nanocomposite catalysts. Qamar M. and colleagues developed N-doped Nb_2_O_5_, utilizing hazardous benzyl alcohol as a solvent and costly metal alkoxides as starting materials to recreate graphene oxide composites for investigating their photoelectrochemical performance in water splitting.^30^ Therefore, it is preferable to synthesize hetero-structured Nb_2_O_5_/graphene using an inexpensive, eco-friendly approach.

Compared with previously reported nanozyme-based H_2_O_2_ sensing platforms, the pyrochlore Fe_2_Nb_2_O_7_ nanozyme exhibits superior analytical performance owing to its unique crystal structure and synergistic Fe–Nb electronic interactions, which promote rapid interfacial electron transfer and efficient H_2_O_2_ activation. The resulting enhanced generation of reactive oxygen species leads to outstanding peroxidase-like activity and a highly sensitive colorimetric response. Unlike many conventional nanozymes that suffer from limited conductivity, insufficient active sites, or poor stability, Fe_2_Nb_2_O_7_ provides abundant accessible catalytic centers, improved charge transport, and excellent structural robustness. EIS analysis further revealed a lower charge-transfer resistance, confirming accelerated electron migration during catalysis. Consequently, the developed sensing platform achieves a low detection limit, wide linear range, excellent reproducibility, and satisfactory recovery in serum samples. These results demonstrate that pyrochlore Fe_2_Nb_2_O_7_ is a stable, enzyme-free, and cost-effective nanozyme with significant potential for practical biosensing and diagnostic applications.

This work reports the first successful synthesis of Fe_2_Nb_2_O_7_ pyrochlore *via* a co-precipitation-assisted route and its unprecedented application as a nanozyme for hydrogen peroxide detection. The study introduces a previously unexplored Fe–Nb pyrochlore platform that combines structural robustness with enzyme-mimicking catalytic activity. The integration of pyrochlore chemistry into peroxide sensing expands the functional scope of mixed-metal oxides and establishes a new material framework for high-performance nanozyme-based analytical systems.

## 2 Experimentation

### 2.1 Reagents and materials

Hydrogen peroxide (H_2_O_2_), iron chloride (FeCl_3_), uric acid (C_5_H_4_N_4_O_3_), potassium permanganate, tyrosine and cysteine were received from Merck. Niobium(v) oxide, ammonia, acetic acid and TMB were received from Sigma-Aldrich. Daejung provided the anhydrous sodium acetate. Lab Scan provided the dimethyl sulfoxide (DMSO). We purchased ascorbic acid and dopamine (C_8_H_11_NO_2_) from Alfa Aesar. BDH was used to create a hydrochloric acid solution. Pellets of sodium hydroxide were obtained from Omicron. Every chemical was used exactly as supplied and met analytical standards. All sample preparation was done using distilled deionized (dI) water.

### 2.2 Characterizations

Surface morphologies of the samples were examined using a variable pressure Field Emission Scanning Electron Microscope (FE-SEM) Apreo S. X-ray diffraction (XRD) analysis was conducted with a Rigaku HyPix-400 MF device. Functional groups in the samples were identified using a Thermo Fisher Scientific Nicolet 6700 Fourier transform infrared spectrometer (FTIR) in attenuated total reflectance (ATR) mode, with a resolution of 8 cm and a scan range of 4000–600 cm^−1^. The Thermo Scientific Multiskan Sky Microplate Spectrophotometer measured absorbance from 200 to 1000 nanometers in 1 nm steps at a scanning speed of 10 seconds.

### 2.3 Synthesis of pyrochlore Fe_2_Nb_2_O_7_

**Scheme 1:**
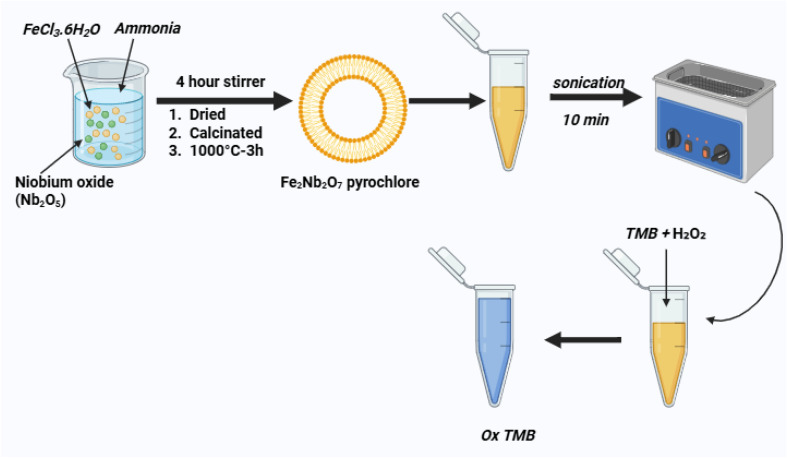
Synthesis of Fe_2_Nb_2_O_7_ pyrochlore with H_2_O_2_ detection.

FeCl_3_·6H_2_O and niobium oxide (Nb_2_O_5_) were mixed in a 1 : 1 ratio in 50 mL of DD water to synthesize novel pyrochlore Fe_2_Nb_2_O_7_. To adjust the pH at 11, ammonia was added, resulting in a white precipitate after stirring for four hours. The stability of the intended metal oxide product is ensured by keeping the pH at a basic level throughout synthesis. Additionally, the precipitation and subsequent formation of the metal oxide are aided by the basic pH. The resulting Fe_2_Nb_2_O_7_ particles were then dried in a hot air oven for 12 hours after being centrifuged with ethanol and DD water to balance the pH. To eliminate additional contaminants, the dried Fe_2_Nb_2_O_7_ particles were calcined for three hours at 1000 °C in an air atmosphere (as shown in Scheme 1).

### 2.4 Colorimetric method for H_2_O_2_ detection

The produced nanomaterials dispersed solid forms were used to evaluate their peroxidase-like catalytic capability. To prepare a uniform and stable dispersion, the dried powders were first converted into well-distributed two-dimensional nanostructures through an ultrasonication-assisted exfoliation process in deionized water, following the method reported in ref. 33. In typical preparation, 2 mg of each dried sample was introduced into 2 mL of distilled water. The mixture was then subjected to continuous ultrasonication for 10 minutes at room temperature. This sonication plays a crucial role in breaking down any aggregated particles, promoting effective separation of the layers, and ensuring that the nanocomposites remain evenly suspended throughout the liquid medium.

The catalytic activity of the homogeneous nanocomposite suspensions was evaluated using a model peroxidase reaction involving hydrogen peroxide (H_2_O_2_) and 3,3′,5,5′-tetramethylbenzidine (TMB) as the chromogenic substrate. Each experiment commenced with the addition of 40 µL of the nanocomposite solution (1 mg mL^−1^) and 40 µL of sodium acetate buffer (100 mM, pH 4) into a reaction tube, followed by gentle shaking for 1 minute to facilitate catalyst equilibration in the acidic medium. Subsequently, 40 µL of H_2_O_2_ solution (1 mM in distilled water) and 40 µL of TMB solution (30 mM in DMSO) were added sequentially.

Upon the introduction of all reactants, a noticeable color change in the reaction mixture was observed. This visible transformation arises due to the oxidation of TMB by H_2_O_2_ catalytically, a process significantly accelerated by the presence of the nanocomposite materials. The appearance and intensity of the color indicate the efficiency of electron transfer facilitated by the catalyst, thereby confirming the peroxidase-mimicking behavior of the synthesized samples. Such a color change serves as a qualitative and quantitative indicator of the catalytic capability of the prepared nanomaterials. With a clear absorption peak at 645 nm, oxidized TMB (Ox TMB) was the cause of this color change in the reaction mixture, influenced by temperature, buffer pH, substrate concentrations (TMB or H_2_O_2_), and catalyst concentration. Optimizing catalytic measurements was essential before identifying H_2_O_2_. The optimizing conditions for the reaction include the amount of nanocomposites, temperature, TMB concentration and pH.

### 2.5 Control trials

By creating a baseline for comparison, a control experiment in colorimetric analysis helps guarantee the precision and dependability of results. It employs a sample treated similarly to the test samples in order to account for any non-specific or background signals but does not include the material being examined. To ascertain the catalytic activity of nanocomposites, the procedure outlined in 2.4 was also employed as a control experiment.

### 2.6 Scavenger hunt tests

In order to investigate the specific role that hydroxyl radicals (˙OH) plays in the catalytic process, a series of controlled radical-scavenging experiments was performed. For this purpose, reaction mixtures were prepared containing Fe_2_Nb_2_O_7_ nanozyme (1 mg mL^−1^), the chromogenic substrate TMB (10 mM), and hydrogen peroxide (H_2_O_2_, 250 µM) in deionized water at room temperature. To selectively quench different reactive species and understand their involvement in the catalytic pathway, three well-known scavengers, isopropyl alcohol (IPA), ethylene diamine tetra-acetic acid disodium salt (EDTA) and ascorbic acid (AA) were each added separately at a concentration of 1 mM. Additionally, an oxygen-removal/O^2−^-related control condition was introduced to evaluate the contribution of oxygen-derived reactive species during the catalytic oxidation process. The corresponding reaction system was treated under oxygen-removal conditions and analyzed alongside the scavenger-treated samples to compare the influence of O^2−^ species on TMB oxidation. To guarantee sufficient interaction between the nanozyme, reactants, and scavengers, the mixtures were allowed to incubate for half an hour. After incubation, the absorbance of each reaction system was measured at 645 nm to determine the extent to which ˙OH radicals contributed to the total catalytic oxidation of TMB.

### 2.7 Selectivity test

To further assess the selectivity and reliability of the sensing platform, additional experiments were conducted in the presence of several common coexisting and potentially interfering analytes. These included ascorbic acid, H_2_O_2_, uric acid, urea, glucose, KCl, NaCl, glycine, cholesterol, dopamine and cysteine. Using the same experimental conditions and procedure described in Section 2.4, each interfering species was individually added to the response system to assess its effect on the sensing response. This approach not only allowed for a systematic examination of the sensor's selectivity toward the target analyte but also provided insight into the recyclability and stability of the nanocomposites under repeated measurement conditions.

### 2.8 Real sample analysis

To further validate the practicality and real-world applicability of the developed H_2_O_2_ sensing platform, its performance was evaluated using human serum samples through a standard spiking recovery approach. Human serum was obtained by Chughtai Lab, Lahore, Pakistan, from three healthy volunteers specifically for analytical testing and used without any processing. After collection, the serum samples were first thawed at room temperature and subsequently stored at 18 °C until used to preserve their biochemical integrity. For clarity, the three samples were labeled as serum sample 1, 2 and 3.

The detection procedure applied to these serum samples followed the same optimized methodology for quantitative H_2_O_2_ measurement in deionized water see Section 2.4. By applying identical experimental conditions, including reagent concentrations and incubation steps, the accuracy and reliability of the sensing system in complex biological matrices could be effectively evaluated. This comparative approach allowed assessment of the sensor's robustness, sensitivity, and suitability for potential clinical or biochemical applications.

### 2.9 Recovery experiment

To evaluate the practical applicability of the proposed method, recovery experiments were performed by spiking human serum samples with known concentrations of H_2_O_2_. The recovery values with the relative standard deviation (RSD) values measured. Furthermore, the obtained results were validated by comparison with a commercial assay kit for the validity and accuracy of the nanozyme.

### 2.10 Determination of LOD and LOQ

The limit of detection (LOD) and limit of quantification (LOQ) were calculated according to the International Conference on Harmonisation (ICH) guideline using the following equations:LOD = 3.3*σ*/*S*andLOQ = 10*σ*/*S*where *σ* is the standard deviation of the blank measurements (or the standard deviation of the response) and *S* is the slope of the calibration curve. These calculations were used to evaluate the sensitivity and quantitative capability of the developed colorimetric sensing platform.^31,32^

### 2.11 Statistical analysis

All experiments were performed at least three times (*n* = 3), and the obtained results are presented as mean ± standard deviation (SD). Statistical analyses were conducted using Origin-Pro 2021. Differences among groups were evaluated using one-way analysis of variance (ANOVA) followed by Tukey's post hoc multiple comparison test for mean separation. A value of *p* < 0.05 was considered statistically significant. Calibration curves were obtained by linear regression analysis, and the correlation coefficient (*R*^2^) was used to assess the goodness of fit. Error bars in all graphical representations indicate the standard deviation of three independent measurements.

## 3 Results and discussion

**Fig. 1:**
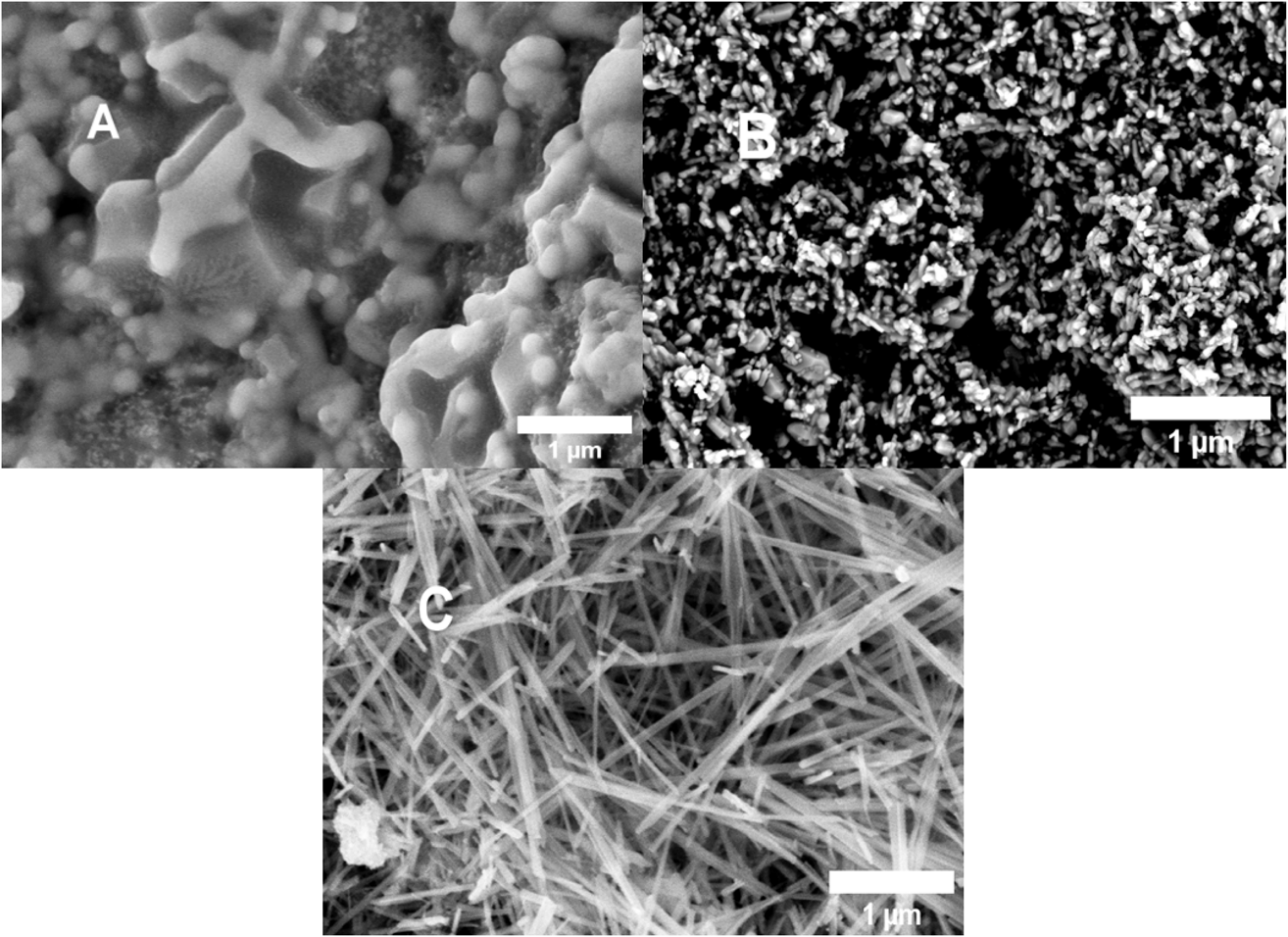
Field-emission scanning electron microscopy (FE-SEM) images illustrating the surface morphology of (A) Nb_2_O_5_, (B) Fe_2_O_3_, and (C) pyrochlore Fe_2_Nb_2_O_7_, highlighting the morphological evolution and structural integration resulting from pyrochlore phase formation.

The Nb_2_O_5_, Fe_2_O_3_, and pyrochlore Fe_2_Nb_2_O_7_ metal oxide nanocomposites' surface morphologies were examined using a variable pressure Field Emission Scanning Electron Microscope (FE-SEM), as illustrated in Fig. 1(A–C).

The micrograph clearly shows that the Nb_2_O_5_ particles have an irregular to quasi-spherical morphology with discernible aggregation. Strong interparticle interactions during the synthesis and post-treatment procedures, as well as the high surface energy of nanoscale metal oxides, are frequently linked to this aggregation tendency. A structure resembling a porous network is formed by the particles' apparent dense packing and linked boundaries. Because it improves the effective surface area, which is essential for catalytic, sensing, and adsorption-based applications, such a morphology is beneficial. Partial agglomeration makes it challenging to identify individual particles, but the overall homogeneity of particle distribution points to regulated nucleation and growth during synthesis. Improved charge transport and electron transfer kinetics may be made possible by interconnected morphology, which is especially advantageous for colorimetric biomarker detection and Nb_2_O_5_ based nanozyme activity.

As can be seen, the Fe_2_O_3_ particles have a shape that is primarily spherical to irregular, with observable aggregation. Iron oxide nanoparticles' strong magnetic dipole–dipole interactions and high surface energy are frequently blamed for this kind of aggregation. While localized clustering indicates partial sintering or particle–particle interactions during drying or calcination, the rather uniform distribution of the particles indicates effective nucleation during synthesis. Because there are many active sites for surface reactions, the particles' rough and textured surface is beneficial for catalytic and sensing applications. Overall, the FESEM results support the prospective use of iron oxide nanostructures in biomarker detection and peroxidase-like catalytic systems by confirming their successful production with a large surface area and appropriate morphology.

The unique fibrous morphology of the synthesized pyrochlore Fe_2_Nb_2_O_7_, which is created by integrating iron oxide and niobium oxide precursors, is seen in the field emission scanning electron microscopy (FESEM) image. Anisotropic crystal development during the pyrochlore phase creation is indicated by the material's observed linked fiber-like nanostructures with elongated and continuous characteristics. The solid-state diffusion and strong Fe–O–Nb bonding interactions that take place during heat treatment, which encourage directed development and structural reorganization, are responsible for the evolution of the fibrous architecture. The successful phase transformation into the pyrochlore Fe_2_Nb_2_O_7_ structure is confirmed by the creation of a one-dimensional fibrous network in contrast to the granular appearance commonly seen in individual iron oxide and niobium oxide phases.

A porous three-dimensional network with interconnected junctions is formed by the fibers' apparent dense entanglement. Because it provides a large number of exposed active sites, a high surface-to-volume ratio and continuous electron transport channels, this shape is especially beneficial. The fibers' rough surface texture increases surface reactivity even more, promoting effective adsorption and catalytic interaction with target analytes. In comparison to traditional nanoparticulate systems, the continuous fibrous framework also helps to improve structural stability and decrease particle agglomeration. Because it improves mass transport, electron transfer kinetics, and catalytic efficiency, this architecture is very advantageous for colorimetric biomarker sensing and nanozyme-based peroxidase-like activity.

**Fig. 2:**
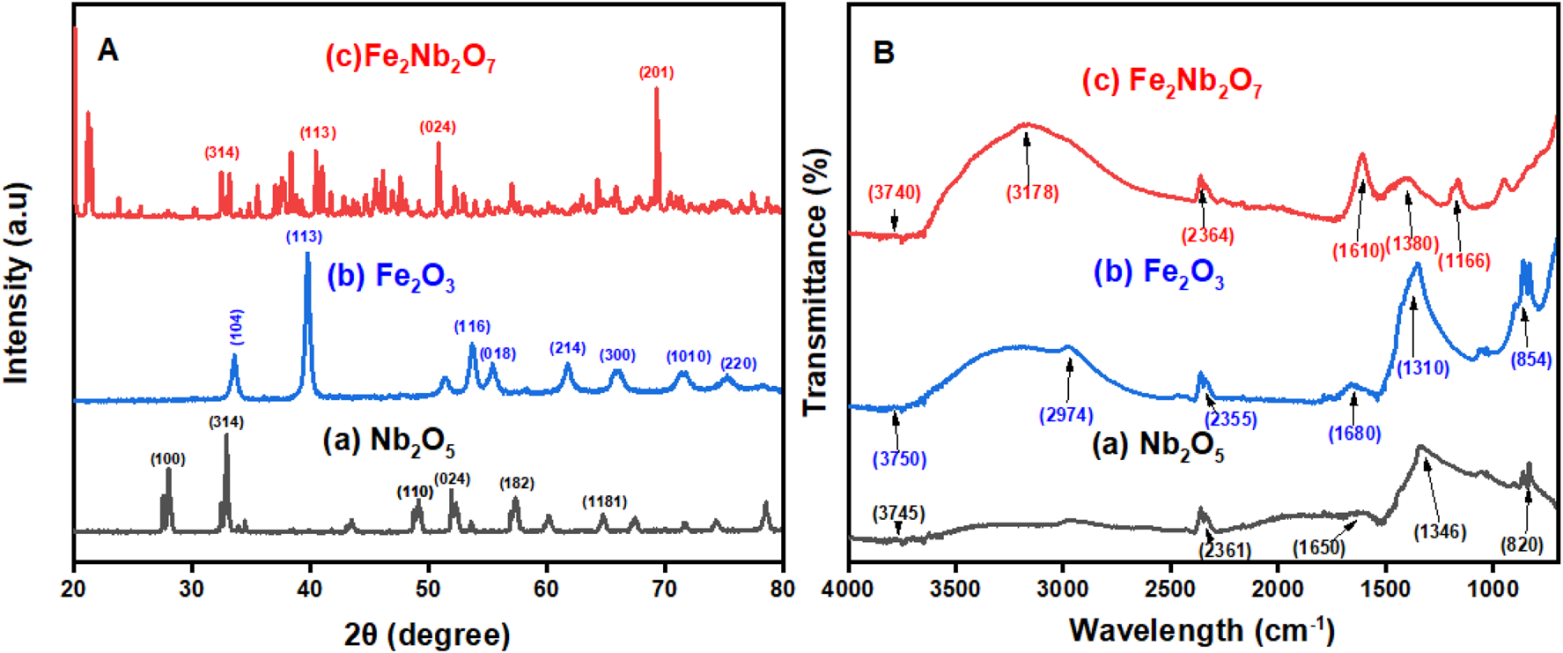
(A) XRD patterns and (B) FTIR spectra of (a) Nb_2_O_5_, (b) Fe_2_O_3_, and (c) pyrochlore Fe_2_Nb_2_O_7_, demonstrating phase purity, crystallographic evolution, and characteristic metal–oxygen bonding, thereby confirming the formation of the Fe_2_Nb_2_O_7_ pyrochlore framework.

Using XRD patterns, the crystallization's nature was determined. Fig. 2(A) displays the XRD patterns for Nb_2_O_5_, Fe_2_O_3_ and pyrochlore Fe_2_Nb_2_O_7_. The Nb_2_O_5_ XRD investigation showed the four peaks. The two-theta (2*θ*) of 28.53°, 32.80°, 49.23°and 57.32° were the locations of the first, second, third and fourth peaks, respectively, and were indexed to (100), (314), (110) and (182) that's correspond to the H–Nb_2_O_5_ structure.^33^

The JCPDS file of α-Fe_2_O_3_ (JCPDS 33-0664) is consistent with the Fe_2_O_3_ XRD analysis, which reveals that the diffraction peaks at 33.0°, 40.7°, 54.0°, 57.6°, 62.3°, 63.9°, 71.8°, and 75.3° were attributed to the (104), (113), (116), (018), (214), (300), (1010), and (220) facets of α-Fe_2_O_3_ nano-polyhedrons, respectively.^34,35^

The pyrochlore Fe_2_Nb_2_O_7_ nanocomposites XRD (X-ray diffraction) assessment showed three noticeable diffraction peaks at 2*θ* values of 32.80°, 40.7°, and 51.94°. The (314), (113), and (024) crystallographic planes were assigned to respective indices to these peaks. The successful integration of Nb_2_O_5_ into the nanocomposite structure is confirmed by the diffraction peak at 32.80°, which corresponds to the (314) plane of Nb_2_O_5_. The peak observed at 40.7°, indexed to the (113) plane, is characteristic of hematite-phase Fe_2_O_3_. These peaks' existence suggests the coexistence of Nb_2_O_5_ and Fe_2_O_3_, suggesting that the composite material retained the individual crystalline characteristics of its components after synthesis.

Fourier Transform Infrared (FTIR) is a technique that can be applied to elucidate the analysis of functional groups over a wide range of wave number ranges, from 400 cm^−1^ to 4000 cm^−1^. Fig. 2(B) displays the FTIR spectra of the Fe_2_Nb_2_O_7_ pyrochlore, Fe_2_O_3_ and Nb_2_O_5_.

The absorbance between 600 cm^−1^ and 1020 cm^−1^ indicates the vibrations of Nb–O–Nb, Fe–O–Nb, and Nb–O bonds in the samples.^36^ The O–H bending vibration in the internal structure of Nb_2_O_5_ is responsible for the band at 1650 cm^−1^.^37^ The peaks at 820, 1346, and 1650 cm^−1^ indicate the presence of Nb_2_O_5_, while the peak at 1706 cm^−1^ originates from carbonate species. The reduced transmittance in the 1600 cm^−1^ to 1780 cm^−1^ range results from the flexural oscillations of O–H, Nb–OH, and Fe–OH bonds due to atmospheric water molecule adsorption in the organized samples.^38^ The bending modes of the water molecule's H–O–H bonds, exhibiting reduced transmittance in the wavenumber range of 2900 cm^−1^ to 3410 cm^−1^, contribute to the stretching vibrations of the nanocomposites.^39^

### 3.1 Peroxidase like activity of nanocomposites

**Fig. 3:**
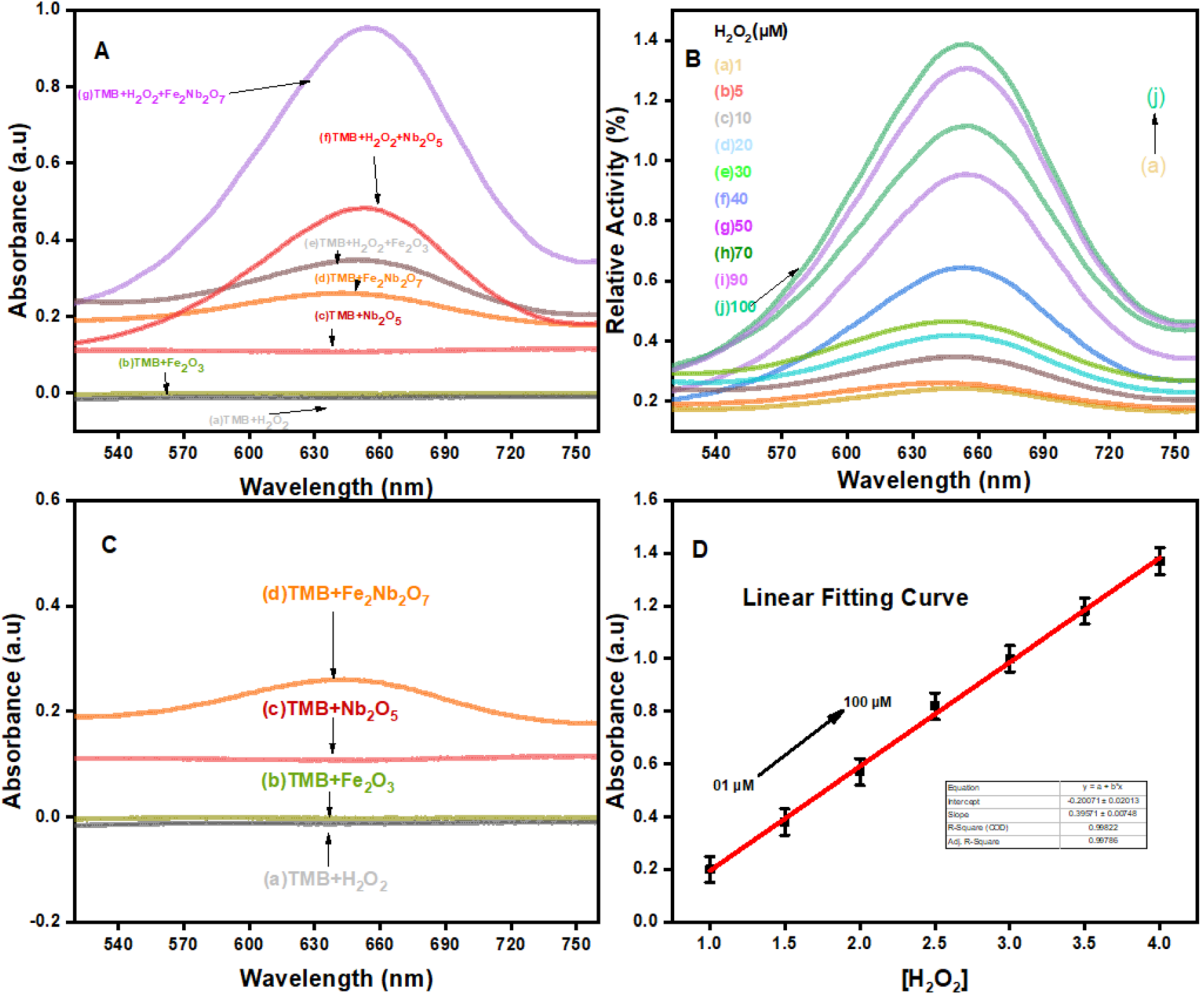
UV-vis absorption analysis of the pyrochlore Fe_2_Nb_2_O_7_ nanozyme for hydrogen peroxide (H_2_O_2_) detection: (A) comparison of absorption spectra of reaction mixtures containing (a) TMB + H_2_O_2_, (b) TMB + Nb_2_O_5_, (c) TMB + Fe_2_O_3_, (d) TMB + Fe_2_Nb_2_O_7_, (e) TMB + H_2_O_2_ + Nb_2_O_5_, (f) TMB + H_2_O_2_ + Fe_2_O_3_, and (g) TMB + H_2_O_2_ + Fe_2_Nb_2_O_7_; (B) typical UV-vis absorption spectra showing catalytic oxidation of TMB in the presence of H_2_O_2_; (C) control experiments validating the specificity of the catalytic system; and (D) corresponding linear calibration plot demonstrating the quantitative detection capability toward H_2_O_2_.

Fig. 3(A) compares the peroxidase-like activity of Nb_2_O_5_, Fe_2_O_3_, and Fe_2_Nb_2_O_7_ in terms of their UV-vis absorption spectra. The interaction of pyrochlore with H_2_O_2_ in the presence of TMB, which resulted in TMB oxidation and reaction mixtures turning blue, was associated with the A645 nm. Fig. 3(A) makes it evident that the reaction mixture of Fe_2_Nb_2_O_7_ + TMB + H_2_O_2_ (curve g) had a greater A645 nm than the reaction systems of Nb_2_O_5_ + TMB + H_2_O_2_ (curve f) and Fe_2_O_3_ + TMB + H_2_O_2_ (curve e).

A peak similar to the Ox TMB absorption peak appears at 652 nm in the presence of glucose, TMB, horseradish peroxidase (HRP), and GOD.^40^ Additionally, an Ox TMB termination solution with a peak extinction coefficient at 450 nm is used to quickly stop the reaction after sufficient development, stabilizing the product for color detection at that wavelength.^41^

The Fe_2_Nb_2_O_7_ reaction system had a higher A645 nm than the Nb_2_O_5_ and Fe_2_O_3_ reaction systems. Compared to Nb_2_O_5_ and Fe_2_O_3_, pyrochlore Fe_2_Nb_2_O_7_ exhibited greater peroxidase-like catalytic activity, which was explained by a higher number of nucleation sites. Additionally, reaction system shows no change, made up of Fe_2_Nb_2_O_7_ + TMB (curve d), TMB + Nb_2_O_5_ (curve c), and TMB + Fe_2_O_3_ (curve b), indicating that no redox reaction took place. These results showed that Fe_2_Nb_2_O_7_ outperformed Nb_2_O_5_ and Fe_2_O_3_ in terms of intrinsic peroxidase-like activity, electron transferability, and charge separation efficiency. These electronic structures enhance electron transport, enabling better mobilization. The absence of peaks at A645 nm and blue coloration when TMB and H_2_O_2_ were mixed without pyrochlore indicates that the catalyst is essential for the reaction to occur.

### 3.2 Optimization of reaction conditions

The reaction conditions included temperature, pH, the quantity of nanocomposites and concentration of TMB were the primary parameters affecting the catalytic activity for the detection of H_2_O_2_. Therefore, it was crucial to optimize these conditions.

**Fig. 4:**
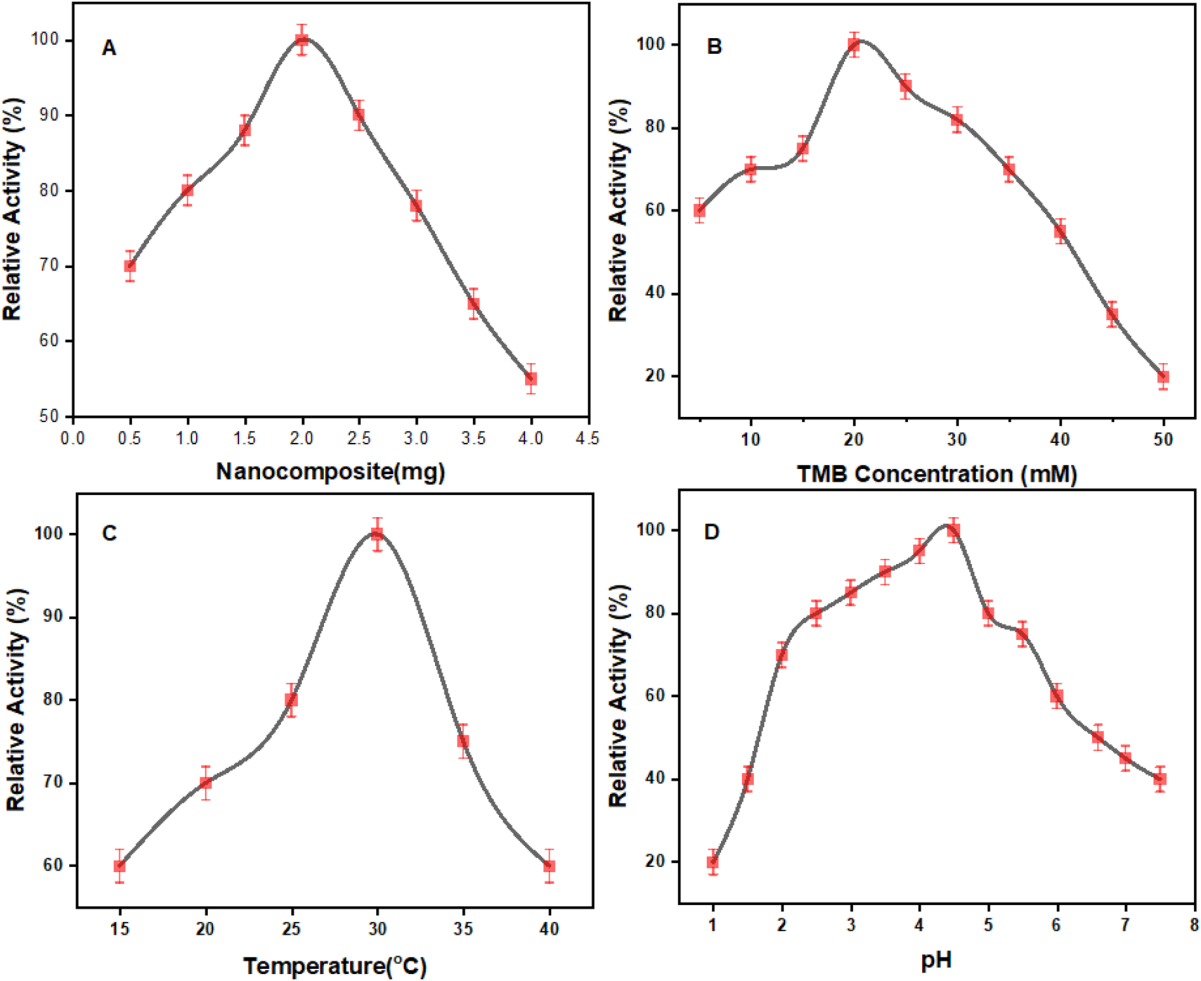
Effects of experimental parameters on the catalytic activity of pyrochlore Fe_2_Nb_2_O_7_ nanocomposites toward hydrogen peroxide detection: (A) influence of nanocomposite loading, (B) TMB concentration, (C) variation with reaction temperature, and (D) dependence on pH, demonstrating the optimal conditions for sensitive performance.

The optimal colorimetric sensing reaction was found by first optimizing the nanoparticle concentration, which was found to be about 2 mg of nanocomposites in 2 mL of distilled water. To put it briefly, the matching test tubes were filled with varying concentrations of nanocomposites while keeping the other parameters constant. No further abnormalities were seen in the colorimetric reaction, even when the amount of nanocomposites was changed. Therefore, as shown in Fig. 4(A), 2 mg was found to be the optimal dosage and employed in further studies. After optimization of nanocomposites, TMB was tested at concentrations from 5 to 50 mM to find the optimal colorimetric response. The best results were achieved with a 20 mM concentration, where the sensor displayed accurate H_2_O_2_ detection due to an adequate substrate amount that balanced sensitivity and noise. Concentrations below or above 20 mM yielded less accurate results, confirming 20 mM as the ideal level for further catalytic processes as seen in Fig. 4(B). At temperatures between 15 °C and 30 °C, the peroxidase-like activity of Fe_2_Nb_2_O_7_ pyrochlore nanocomposites increased, as higher temperatures enhance the diffusion and kinetic energy of H_2_O_2_ molecules. However, catalytic efficacy dropped sharply at temperatures above 40 °C, indicating that the optimal temperature for catalytic activity is 30 °C as seen in Fig. 4(C).^42,43^ In the meantime, another significant element influencing catalytic effectiveness is pH. The study examines pH impact on the catalytic activity of Fe_2_Nb_2_O_7_ pyrochlore nanocomposites, revealing an increase in activity from pH 1 to 4.5, followed by a decrease at pH 7.5. The architecture of the nanocomposites provides an ideal 4.5 pH for catalytic sites, demonstrating that stable catalytic activity occurs in mildly acidic conditions.^44,45^

### 3.3 Detection of H_2_O_2_

Fe_2_Nb_2_O_7_ nanocomposites were synthesized to assess different H_2_O_2_ levels under optimal conditions. The evaluation employed a sensitive colorimetric method, linking H_2_O_2_ concentration to absorption strength at 645 nm. The newly built sensor's ability to detect H_2_O_2_ has been tested using a range of H_2_O_2_ concentrations. Fig. 3(B) illustrates how colorimetric sensors react to different H_2_O_2_ levels. The peak intensity and sensitivity of the sensor were both high at lower H_2_O_2_ concentrations, but they decreased linearly as the H_2_O_2_ concentration increased. The total *R*^2^ value of this approach is 0.998, and its linear range for H_2_O_2_ detection is 01–100 µM. The limit of quantification (LOQ) was 1.88 µM and the limit of detection (LOD) was 0.62 µM, as shown in Fig. 3(C). The recommended colorimetric technology highlights its sensitivity in detecting H_2_O_2_ and has benefits like direct eye observation, low cost, and a low detection limit.

### 3.4 Control experiments

The control experiment revealed colorimetric changes associated with the prepared pyrochlore, as no changes were observed in TMB + H_2_O_2_, TMB + Nb_2_O_5_, and TMB + Fe_2_O_3_. The lack of an absorption peak or color shift without the pyrochlore confirms its role in the observed color change of TMB (as seen in Fig. 3(C)).

### 3.5 Scavenger experiments

**Fig. 5:**
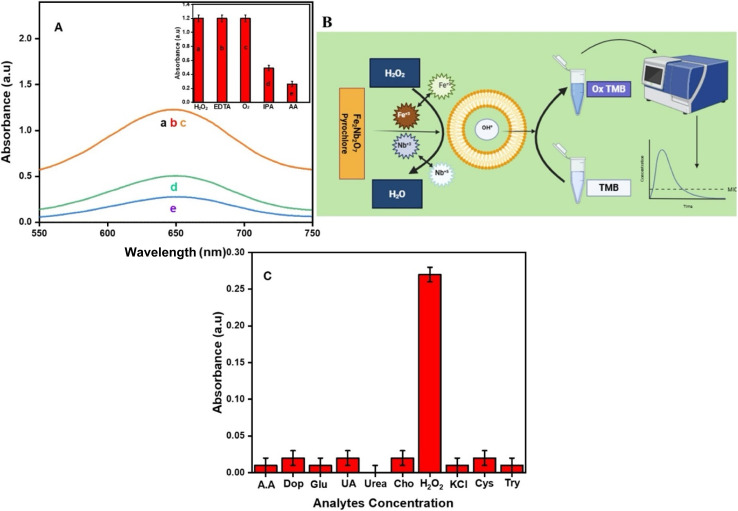
(A) UV spectrum of different scavengers: (a) H_2_O_2_ (b) EDTA (c) O^2−^ (d) IPA (e) ascorbic acid added to the Fe_2_Nb_2_O_7_ + H_2_O_2_ + TMB reaction system, corresponding absorbance response shows in the inset (B) proposed mechanism for the detection of H_2_O_2_ through novel pyrochlore Fe_2_Nb_2_O_7_ (C) the response of Fe_2_Nb_2_O_7_ + TMB system towards H_2_O_2_ and various interferences.

To learn more about the catalytic process, the impact of several quenchers on the Fe_2_Nb_2_O_7_ + H_2_O_2_ + TMB reaction system was investigated. Superoxide anion radicals (O^2−^), hydroxyl radicals (˙OH), and electron hole pairs (h^+^) are potential intermediary reactive species generated in the catalytic reaction. EDTA, or ethylenediaminetetraacetic acid disodium salt, is frequently employed to show that h^+^ species exist.^46^ The addition of EDTA did not affect the reaction system's absorbance at 645 nm (Fig. 5(A-b)). Additionally, when oxygen was eliminated from the reaction system, absorbance remained unchanged, indicating that O^2−^ had no impact on catalysis (c).^46^

Isopropanol (IPA) is commonly used as a trapping agent to effectively remove ˙OH from the solution.^47^ When IPA was added, the absorbance of the reaction system dropped, indicating that ˙OH was the primary active species in the catalytic activity (d). Additionally, the peroxidase activity was evaluated using the natural antioxidant ascorbic acid's (AA) inhibiting impact.^48,49^ As expected, the oxidation rate of TMB was significantly reduced by the addition of AA (e) as compared to the control. This work showed that the peroxidase-like activity of novel pyrochlore Fe_2_Nb_2_O_7_ was caused by the ˙OH radicals, which were necessary for the oxidation of TMB.

### 3.6 Mechanistic investigation of peroxidase-like activity

The participation of reactive oxygen species, specifically ˙OH radicals, in the catalytic activity of Fe_2_Nb_2_O_7_ nanozyme can be explained by spectroscopic studies and mechanistic studies of Fe/Nb-based nanozymes. The FTIR study shows that there is an abundance of hydroxyl group at the surface, which is shown by the wide stretching band observed at the frequency associated with adsorbed water molecules and terminal hydroxyl groups. This type of functional group on the surface of metal oxides is known to play an important role in the adsorption and dissociative oxidation of hydrogen peroxide. They help in the decomposition of hydrogen peroxide to yield highly reactive intermediates.

In addition, studies on the mechanisms of iron-based nanozymes have repeatedly confirmed that iron–oxygen complexes promote redox switching between Fe^2+^ and Fe^3+^ forms, thus facilitating H_2_O_2_ decomposition through Fenton-type chemistry and generating ˙OH as the main reactive species. Likewise, Nb-containing oxides have been shown to boost catalytic efficiency owing to electron density control and improved charge transfer at the interface facilitated by Nb–O interactions, resulting in accelerated ROS generation. Synergism between Fe and Nb in mixed-metal pyrochlores leads to increased electron delocalization and surface redox activity, correlating directly with superior peroxidase-like activity.

Therefore, in combination with known Fe Fenton chemistry, Nb-based electronic tuning, and the FTIR-verified presence of hydroxyl groups on the nanozyme surface, it can be argued that TMB oxidation occurs mainly through a hydroxyl-mediated reaction scheme.

### 3.7 The proposed mechanism for H_2_O_2_ detection

The colorimetric detection of H_2_O_2_ using the pyrochlore Fe_2_Nb_2_O_7_ nanozyme operates through a sophisticated peroxidase-mimetic catalytic cycle, leveraging the unique electronic structure of its mixed-metal oxide lattice. The mechanism can be dissected into two concurrent and synergistic half-reactions that constitute a classic peroxidase-like cascade. First, the peroxide reduction half-reaction occurs where hydrogen peroxide acts as the oxidizing agent. The surface Fe^2+^/Fe^3+^ redox couples on the Fe_2_Nb_2_O_7_ nanozyme facilitate a heterogeneous Fenton-like process, decomposing H_2_O_2_ into hydroxyl radicals while the iron itself is oxidized.

1 Fe^2+^ (surface) + H_2_O_2_ → Fe^3+^ (surface) + ˙OH + ˙OH

The generated hydroxyl radical (˙OH) is an extremely potent and non-specific oxidant. Simultaneously, the chromogen oxidation half-reaction takes place. A colorless chromogenic substrate, such as 3,3′,5,5′-tetramethylbenzidine (TMB), adsorbs onto the nanozyme surface and donates an electron, becoming oxidized. In its one-electron oxidized state, TMB forms a blue-colored charge-transfer complex (Ox TMB).

2 TMB (colorless) → TMB^+^ (blue) + e^−^

The critical role of the Fe_2_Nb_2_O_7_ nanozyme is to bridge these two half-reactions by efficiently shuttling electrons. The Fe^3+^ formed in reaction (1) is reduced back to its Fe^2+^ state by accepting the electron released from TMB in reaction (2), thereby closing the catalytic cycle and regenerating the active site.

3 Fe^3+^ (surface) + e^−^ (from TMB) → Fe^2+^ (surface)

This regenerative step is crucial for sustaining the catalytic turnover. The niobium species (Nb^3+^/Nb^5+^) within the robust pyrochlore lattice (A_2_B_2_O_7_) plays a pivotal role in regulating the catalytic activity; the coexistence of different oxidation states provides reversible redox flexibility and enhances electron-transfer processes. The high oxidation state of Nb^5+^ and the electron-donating capability of Nb^3+^ help modulate the electron density on the iron centers, promoting H_2_O_2_ and TMB adsorption, stabilizing reactive intermediate species, and facilitating faster interfacial electron transfer kinetics during the catalytic reaction.

The overall net reaction is the nanozyme-catalyzed oxidation of TMB by H_2_O_2_:(Overall) 2H_2_O_2_ + TMB (colorless) → 4H_2_O + Ox TMB (blue)

The concentration of ˙OH radicals produced, which is determined by the starting H_2_O_2_ concentration, is directly correlated with the intensity of the ensuing blue hue. Pyrochlore Fe_2_Nb_2_O_7_ is positioned as a potent inorganic replacement for the natural enzyme horseradish peroxidase (HRP) because to its measurable color shift, it forms the basis of a colorimetric assay that is extremely sensitive and selective (as illustrated in Fig. 5(B)).

### 3.8 The selectivity of the sensor

In assessing the selectivity of H_2_O_2_ detection using Fe_2_Nb_2_O_7_ pyrochlore as a peroxidase mimic, the absorbance responses of various coexisting chemicals cholesterol, glucose, KCl, uric acid, hydrogen peroxide, urea, ascorbic acid, NaCl, dopamine, glycine, and cystine were evaluated, revealing poor absorbance for uric acid, glucose, cholesterol, dopamine, KCl, urea, glycine, ascorbic acid, NaCl, and cysteine as can be shown in Fig. 5(C). Despite the presence of interfering chemicals, no significant absorbance changes were observed, indicating that high absorbance is only achievable with H_2_O_2_. The findings confirm the selectivity of H_2_O_2_ detection, as these substances did not affect the results.

### 3.9 Detection of H_2_O_2_ in real sample

**Fig. 6:**
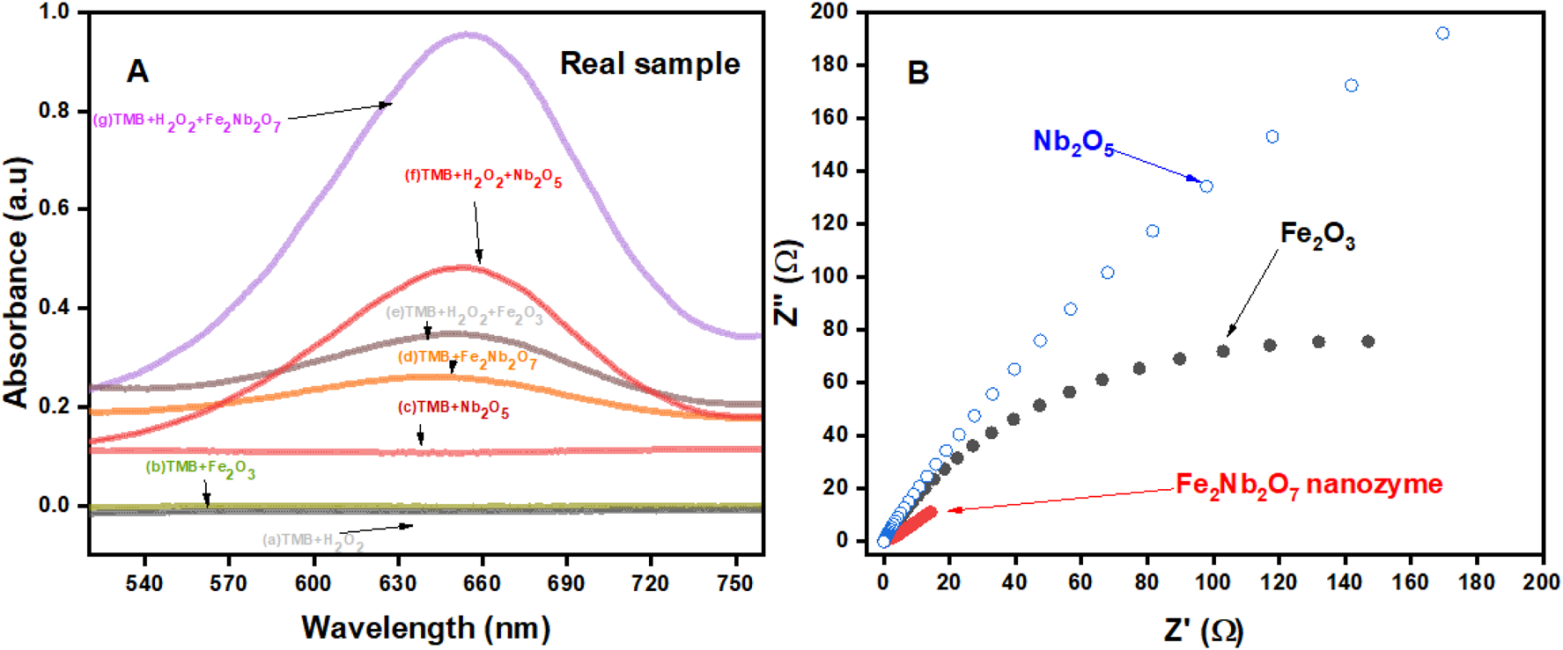
(A) Detection of H_2_O_2_ in real sample (B) electrochemical Impedance Spectroscopy (EIS) Nyquist plots of the reference sample and the nanozyme.

A UV-vis spectrophotometer was used to examine three serum samples for colorimetric changes of H_2_O_2_. Colorimetric measurement showed that the solutions turned blue after two minutes as the H_2_O_2_ content rose. An absorption peak at 645 nm was seen, and it rose linearly with the H_2_O_2_ concentration, much as the detection method in DI water (as seen in Fig. 6(A)).

### 3.10 Electrochemical impedance analysis (EIS)

Fig. 6(B) represents the Nyquist plot for Nb_2_O_5_, Fe_2_O_3_, and Fe_2_Nb_2_O_7_ pyrochlore nanozyme using Electrochemical Impedance Spectroscopy (EIS). The plot gives information about the properties of charge transfer from the material under study. Amongst all three materials, Fe_2_Nb_2_O_7_ pyrochlore nanozyme possesses a smaller semicircle as compared to Nb_2_O_5_ and Fe_2_O_3_, showing that it has much less charge transfer resistance (*R*_ct_) and excellent electron transfer properties across the interface. Moreover, Nb_2_O_5_ showed high impedance values, implying low conductivity, whereas the case with Fe_2_O_3_ was an intermediate one. This is because of the synergistic effect of Fe and Nb in the pyrochlore structure. The improved electron transport in Fe_2_Nb_2_O_7_ is attributed to the synergistic interaction between Fe and Nb within the pyrochlore framework, which facilitates efficient charge migration and supports its superior peroxidase-like catalytic activity.

### 3.11 Recovery experiment

To evaluate the practical applicability of the proposed method, recovery experiments were performed by spiking human serum samples with known concentrations of H_2_O_2_. The recoveries were found to be in the range of 96.0% to 102.0%, with relative standard deviation (RSD) values below 2.4 to 3.6%, indicating good accuracy and reproducibility. Furthermore, the results obtained were validated by comparison with a commercial assay kit, showing excellent agreement between the two methods. These results confirm the reliability of the proposed nanozyme-based sensor for H_2_O_2_ detection in complex biological media (Table 1).

**Table 1:** Recovery study and comparison of proposed Fe_2_Nb_2_O_7_ nanozyme method with commercial assay kit in human serum samples

Sample	Spiked (µM)	Proposed method (µM)	Commercial kit (µM)	Recovery (%)	RSD (%)
Serum 1	10	9.6	9.4	97	3.2
Serum 1	50	51.2	49.5	102.4	2.7
Serum 2	10	10.4	10.1	103.5	3.5
Serum 2	50	49.6	49.2	98.3	2.6
Serum 3	10	8.9	9.2	97.9	3.8
Serum 3	50	51.3	50.6	101.2	2.9

### 3.12 Comparison with previously reported nanozymes

Table 2 summarizes the analytical performance of the developed pyrochlore Fe_2_Nb_2_O_7_ nanozyme and compares it with previously reported peroxidase-mimicking nanomaterials for H_2_O_2_ detection. The Fe_2_Nb_2_O_7_ nanozyme exhibited an excellent linear response over the concentration range of 1–100 µM with an ultra-low limit of detection (LOD) of 0.62 µM. Compared with Se-g-C_3_N_4_ (LOD = 1.6 µM), MeSe-g-C_3_N_4_ (LOD = 1.8 µM), and Fe_3_O_4_/lignin nanoparticles (LOD = 2 µM), the proposed nanozyme demonstrates significantly enhanced sensitivity toward H_2_O_2_. Furthermore, its detection capability surpasses that of ZnS–MMT nanocomposites (10.48 µM), FePt–Au nanoparticles (12.33 µM), N-GQDs (16 µM), Ag_2_S–MMT nanocomposites (19.16 µM), and Cu_2_(OH)_3_Cl–CeO_2_ nanocomposites (50 µM), highlighting its superior catalytic efficiency.

**Table 2:** Comparison of linear ranges, LODs of different nanomaterials as peroxidase mimetic for H_2_O_2_ assays

Sensing materials	Linear range (µM)	LOD (µM)	References
Pyrochlore Fe_2_Nb_2_O_7_	01–100	0.62	Present work
Se-g-C_3_N4	16–4000	1.6	50
MeSe-g-C_3_N_4_	18–18000	1.8	45
Fe_3_O_4_/lignin NPs	5–100	2	51
ZnS-MMT nanocomposites	70–600	10.48	52
FePt–Au NPs	20–700	12.33	53
N-GQDs color	25–375	16	54
Ag_2_S-MMT nanocomposites	200–1200	19.16	55
Cu_2_(OH)_3_Cl–CeO_2_	100–2000	50	56

Although some previously reported materials exhibit wider linear ranges extending into the millimolar region, such as MeSe-g-C_3_N_4_ (18–18 000 µM) and Se-g-C_3_N_4_ (16–4000 µM), the Fe_2_Nb_2_O_7_ nanozyme offers markedly lower detection limits, making it particularly suitable for trace-level H_2_O_2_ analysis. The outstanding analytical performance can be attributed to the unique pyrochlore crystal structure, which promotes efficient electron transfer between Fe and Nb active centers and facilitates rapid generation of reactive oxygen species during the catalytic decomposition of H_2_O_2_. In addition, the synergistic interaction between Fe and Nb species provides abundant accessible catalytic sites, thereby enhancing peroxidase-like activity and signal amplification. Therefore, the proposed Fe_2_Nb_2_O_7_ nanozyme demonstrates competitive and, in many aspects, superior sensing performance compared with previously reported nanozyme-based H_2_O_2_ sensing platforms, indicating its strong potential for practical colorimetric biosensing and environmental monitoring applications.

## 4 Conclusion

In conclusion, we have created novel Fe_2_Nb_2_O_7_ pyrochlore using the co-precipitation method with iron chloride and niobium(v) oxide. Niobium oxide benefited from the addition of Fe_2_O_3_, which increased the surface area of novel pyrochlore Fe_2_Nb_2_O_7_ and improved porosity. By promoting effective electron transfer at the heterojunction interface, the synergistic integration of iron oxide and niobium oxide greatly increases peroxidase-like catalytic activity, resulting in quicker reaction kinetics and enhanced sensitivity toward H_2_O_2_.

Iron oxide offers a large number of redox-active Fe^2+^/Fe^3+^ centers that efficiently catalyze the breakdown of H_2_O_2_, whereas niobium oxide offers strong metal–support connections, good chemical stability, and more surface oxygen vacancies that encourage the adsorption and activation of H_2_O_2_ molecules. Superior signal amplification, low detection limits, and a broad linear response range are the outcomes of this combination. Furthermore, the composite nanozyme is extremely dependable for H_2_O_2_ sensing in complex matrices due to its great stability against pH and temperature fluctuations, remarkable repeatability, and resistance to interference from common coexisting species. The development of a colorimetric assay for H_2_O_2_ utilizes the reaction between TMB and H_2_O_2_ in the presence of Fe_2_Nb_2_O_7_ pyrochlore, resulting in a color change to blue. The novel pyrochlore Fe_2_Nb_2_O_7_ nanozyme is inexpensive, easy to produce, and retains catalytic performance over time, making it a promising enzyme-free peroxidase mimic for biosensing and medical diagnostics.

Despite these promising results, the present study is primarily demonstrated under controlled laboratory conditions. Potential matrix effects in complex biological samples, along with long-term stability, batch-to-batch reproducibility, and scalability of synthesis, remain as key limitations that warrant further investigation prior to practical clinical translation.

Future work should focus on improving selectivity in real biological environments and extending the platform toward multiplexed and portable sensing applications, particularly for point-of-care diagnostics. In addition, integration with user-friendly readout systems, such as paper-based devices and smartphone-assisted quantification, could significantly enhance practical applicability. Further mechanistic insights into pyrochlore charge transfer and catalytic pathways, supported by advanced spectroscopic and theoretical studies, would also be valuable for rational design of next-generation nanozyme systems.

## Author contributions

All authors have contributed and approved the final version of the manuscript.

## Conflicts of interest

The authors declare no competing financial interest.

## Data Availability

The authors confirm that the data supporting the findings of this study are available within the article.
